# Study on the differential hepatotoxicity of raw polygonum multiflorum and polygonum multiflorum praeparata and its mechanism

**DOI:** 10.1186/s12906-024-04463-9

**Published:** 2024-04-17

**Authors:** Chaowen Huang, Yu Jiang, Qing Bao, Lu Wang, Lin Tang, Yanjuan Liu, Lei Yang

**Affiliations:** 1 Department of Preparations, the First Hospital of Hunan University of Chinese Medicine, Changsha City, China; 2 Institute of Emergency Medicine, Hunan Provincial People’s Hospital, The First Affiliated Hospital of Hunan Normal University, 69 Jiefang Western Road, Changsha City, 410000 Hunan China

**Keywords:** Polygonum multiflorum, Hepatotoxicity, Raw polygonum multiflorum, Polygonum multiflorum praeparata, Ferroptosis

## Abstract

**Background:**

Polygonum multiflorum (PM), a widely used traditional Chinese medicine herb, is divided into two forms, namely raw polygonum multiflorum (RPM) and polygonum multiflorum praeparata (PMP), according to the processing procedure. Emerging data has revealed the differential hepatotoxicity of RPM and PMP, however, its potential mechanism is still unclear.

**Methods:**

In our study, we investigated the differential hepatotoxicity of RPM and PMP exerted in C57BL/6 mice. First, sera were collected for biochemical analysis and HE staining was applied to examine the morphological alternation of the liver. Then we treated L02 cells with 5 mg / mL of RPM or PMP. The CCK8 and EdU assays were utilized to observe the viability and proliferation of L02 cells. RNA sequencing was performed to explore the expression profile of L02 cells. Western blotting was performed to detect the expression level of ferroptosis-related protein. Flow cytometry was used to evaluate ROS accumulation.

**Results:**

In our study, a significant elevation in serum ALT, AST and TBIL levels was investigated in the RMP group, while no significant differences were observed in the PMP group, compared to that of the CON group. HE staining showed punctate necrosis, inflammatory cell infiltration and structural destruction can be observed in the RPM group, which can be significantly attenuated after processing. In addition, we also found RPM could decrease the viability and proliferation capacity of L02 cells, which can be reversed by ferroptosis inhibitor. RNA sequencing data revealed the adverse effect of PM exerted on the liver is closely associated with ferroptosis. Western blotting assay uncovered the protein level of GPX4, HO-1 and FTL was sharply decreased, while the ROS content was dramatically elevated in L02 cells treated with RPM, which can be partially restored after processing.

**Conclusions:**

The hepatotoxicity induced by RPM was significantly lower than the PMP, and its potential mechanism is associated with ferroptosis.

**Supplementary Information:**

The online version contains supplementary material available at 10.1186/s12906-024-04463-9.

## Introduction

Polygonum multiflorum (PM), a well-known traditional Chinese medicine, has been commonly used to treat fatty liver, hyperlipidemia, tumors, learning and memory impairment and Parkinson’s diseases [1]. According to the processing procedure, PM is divided into two forms: raw polygonum multiflorum (RPM) and polygonum multiflorum praeparata (PMP) [2]. However, the administration of PM causes a number of adverse effects, among which hepatotoxicity is the most common [3–5]. The processing procedure could decrease the hepatotoxicity of PM, and PMP products are considered to be relatively safe compared with RPM products [6, 7]. Previous reports have analyzed hepatotoxic components in PM, however, the toxic mechanisms of PM require further study.

Ferroptosis is a recently discovered type of cell death that is an oxidative cell death depending on iron ion and induced by small molecules [8, 9]. It occurs as a result of an imbalance between intracellular production and the degradation of lipid-based reactive oxygen species (ROS) [10, 11]. Ferroptosis inducers act directly or indirectly on glutathione peroxidase 4 (GPX4) through different pathways, resulting in cell antioxidant capacity decrease, ROS accumulation, and ultimately oxidative cell death [12–14]. Numerous studies have shown that ferroptosis is closely associated with liver damage, with the amount of iron ions and lipid-based ROS increased in the liver lesions [15–18]. However, it is not clear whether PM induced liver injury is related to ferroptosis.

In this study, we first experimentally evaluated the hepatotoxic effects of RPM and PMP products. Then, we explored the underlying molecular pathways involved in PM induced hepatotoxicity through a transcriptomic approach. Subsequently, we validated that PM-induced hepatotoxicity was regulated by ferroptosis. All of these results provided new insights into PM-induced hepatotoxicity.

## Materials and methods

### Medicine process

The root of PM were purchased from the First Affiliated Hospital of Hunan University of Traditional Chinese Medicine, in accordance with the standards established by the Chinese Pharmacopoeia, which was identified by Zhiguo Zhang, a professor at the first hospital of Hunan University of Chinese Medicine. RPM product: polygonum multiflorum (unprocessed), PMP product: polygonum multiflorum was soaked and steamed in black bean juice for 4 h, drying for 4 h. The above procedure was repeated nine times. After the processing procedure, RPM and PMP were crushed and extracted with 70% ethanol. They were extracted by heating under reflux using 70% ethanol for twice, with a 10-fold amount of the medicine for the first time and a 8-fold amount of the medicine for the second time. Then, the isolated extracts were combined and concentrated under reduced pressure and the concentrated medicine was obtained.

### Animals

C57BL/6 mice (6–8 weeks) were purchased from SJA Laboratory Animal Co. Ltd. (Changsha, Hunan, China), and housed in a specific pathogen-free environment at 25 °C with a 12:12 h day/night cycle. All mice were randomly divided into three groups: control group (CON): gavaged with saline; RPM group: gavaged with RPM (9 g/kg); PMP group : gavaged with processed PMP (9 g/kg). After 30 days, the mice were anesthetized with 2% isoflurane and sacrificed by cervical dislocation. All methods are reported by ARRIVE guidelines (https://arriveguidelines.org) for the reporting of animal experiments. All protocols performed in our study were approved by the Medical Ethics Committee of the Hunan Provincial People’s Hospital (Grant No.2021 − 168).

### Sample collection

Serum and liver tissues were collected and stored at -80 °C for further analysis.

### Biochemical analysis

Serum levels of were analyzed using an automated biochemistry instrument (Biobase).

### Hematoxylin-eosin staining

The liver tissues from the mice was quickly collected and fixed with 4% paraformaldehyde for 24 h, according to the standard protocol. Subsequently, the 5 mm thick sections were embedded into the paraffin. Hematoxylin-eosin (HE) staining was then applied to examine the change of morphology in liver tissues. Finally, these stained sections were observed under an optical microscope to detect the alternation of tissue morphology.

### Cell viability assay

L02 human liver cell line was purchased from the Cell Bank of China Academy of Sciences (Shanghai, China). The L02 human liver cell line was cultured in 1640 complete medium containing 10% fetal bovine serum (FBS) and 1% Penicillin/streptomycin at 37 °C in an environment of 5% CO_2_. The cytotoxicity of RPM or PMP exerted on the L02 cell was determined by the CCK8 assay. 5 × 10^3^ cells were seeded in 96-well plates and cultured for 24 h, followed by treatment with different concentrations of RPM or PMP (0, 1.25, 2.5, 5, 10, and 20 mg/ml). After a 24 h treatment, 10ul of CCK8 (Beyotime, China, Cat NO: C0037) reagents was added to each well, and the cells were further cultured for 1.5 h. Lastly, the absorbance was measured at 450 nm using the Elx808 microplate reader (Biotek).

### EdU proliferation assay

5 × 10^4^ L02 cells were seeded in 12-well plates and cultured in complete culture medium supplemented with 10% FBS, followed by treatment with different concentrations of RPM or PMP for 24 h. The EdU proliferation assay was then used to examine the proliferation capacity of L02 cells, according to the manufacturer’s instructions. Firstly, the cells were incubated with 10µM EdU (Cellorlab, China, Cat NO: CX002) for 2 h. Then remove the culture medium and fix with 4% paraformaldehyde. Subsequently, cells were permeabilized with Triton X-100 and 500µL Click additive solution was added. 30 min later, 500µL DAPI (1X) solution was added and the fluorescence intensity was measured using the fluorescent microscope at 40 magnification.

### RNA sequencing

5 × 10^5^ L02 cells were seeded in the six-well plates and cultured for 24 h at 37℃ in an environment of 5% CO_2_, followed by the treatment with 5 mg/mL of RPM or PMP for 24 h. After completion, remove the culture medium and wash the cells twice with cold PBS. Using TRizol, total RNA was extracted and harvested. Then, the concentration and purity were tested using NanoDrop 2000 (Thermo Fisher Scientific, Wilmington, DE). RNA integrity was assessed using the Agilent Bioanalyzer 2100 RNA Nano 6000 Assay Kit (Agilent Technologies, CA, USA). 1 µg RNA was utilized for sequencing. Subsequently, sequencing libraries were generated using the NEBNext UltraTM RNA Library Prep Kit for Illumina (NEB, USA) following the manufacturer’s recommendation. The clustering of the index-coded samples was performed on a cBot Cluster Generation System using TruSeq PE Cluster Kit v4-cBot-HS (Illumia) according to the manufacturer’s instructions. After cluster generation, library preparations were sequenced on an Illumina platform, and paired-end reads were generated. The raw reads were further processed with a bioinformatic pipeline tool, BMKCloud (www.biocloud.net) online platform. Raw data (raw reads) of fastq format were processed through in-house Perl scripts. Gene function was annotated on the basis of the following databases: Nr (NCBI nonredundant protein sequences); Nt (NCBI nonredundant nucleotide sequences); Pfam (Protein family); KOG/COG (Clusters of orthologous groups of proteins); Swiss-Prot (A manually annotated and reviewed protein sequence database); KO (KEGG Ortholog database) [19]; GO (Gene Ontology). Differential expression analysis of two conditions/groups was performed using the DESeq2.

### Reactive oxygen species (ROS) content detection

5 × 10^5^ L02 cells were seeded in the six-well plates and cultured for 24 h at 37℃ in an environment of 5% CO_2_, followed by the treatment with 5 mg/mL of RPM or PMP for 24 h. After completion, remove the culture medium and incubate the cells with incomplete culture medium containing 5 μm DCFH-DA (Beyotime, China, Cat NO: S0033S) for 30 min. Subsequently, wash the cells three times with incomplete culture medium. Finally, cells were resuspended in PBS after digested with tyrpsin and the ROS content was detected by flow cytomery (CytoFLEX, Beckman). The acquired data was analyzed using FlowJo V10.

### Protein preparation and western blotting

The radioimmunoprecipitation assay (RIPA) was used to extract and harvest proteins from L02 cells, and the BCA assay was used to measure protein concentration. Total protein (20 µg per lane) was separated on 10% or 12% sodium dodecyl sulfate-polyacrylamide gel electrophoresis (SDS-PAGE) and transferred to a polyvinylidene fluoride (PVDF) membrane. Then the PVDF membrane was soaked in 5% skim milk for 1.5 h, which was dissolved in Tris buffered saline Tween (TBST). Subsequently, the soaked PVDF membrane was incubated overnight at 4 °C with a rabbit antibody against GPX4 (Affinity, China, Cat NO: AF5384), HO-1(Affinity, China, Cat NO: AF5393), FTL (Abclonal, China, Cat NO: A11241), GSS (Abclonal, China, Cat NO: A5652) and GAPDH (Cell Signaling Technology, USA, Cat NO: 4970 S) at a dilution of 1:1000, respectively. After completion, the membrane was washed with TBST three times and incubated with a secondary anti-rabbit antibody (Abclonal, China, Cat NO: AS014) for 1 h at room temperature. Similarly, the membrane was washed with TBST three times again. Finally, specific bands were visualized using a chemiluminescent substrate and detected using the Omega Lum C Gel Imaging System (Bio-rad). Protein band intensity was measured and quantified by ImageJ software.

### Statistical analysis

All data were presented with mean ± s.d and statistic analyzed by the GraphPad Prism 8.0 software. The Student’s unpaired *t-test* was used to determine statistical significance between the two groups. A two-way analysis of variance was applied to compare the significant difference among multiple groups. *P* < 0.05 was considered statistically significant.

## Results

### An increased liver toxicity was observed in the RPM group compared to that of the PMP group

**Fig. 1:**
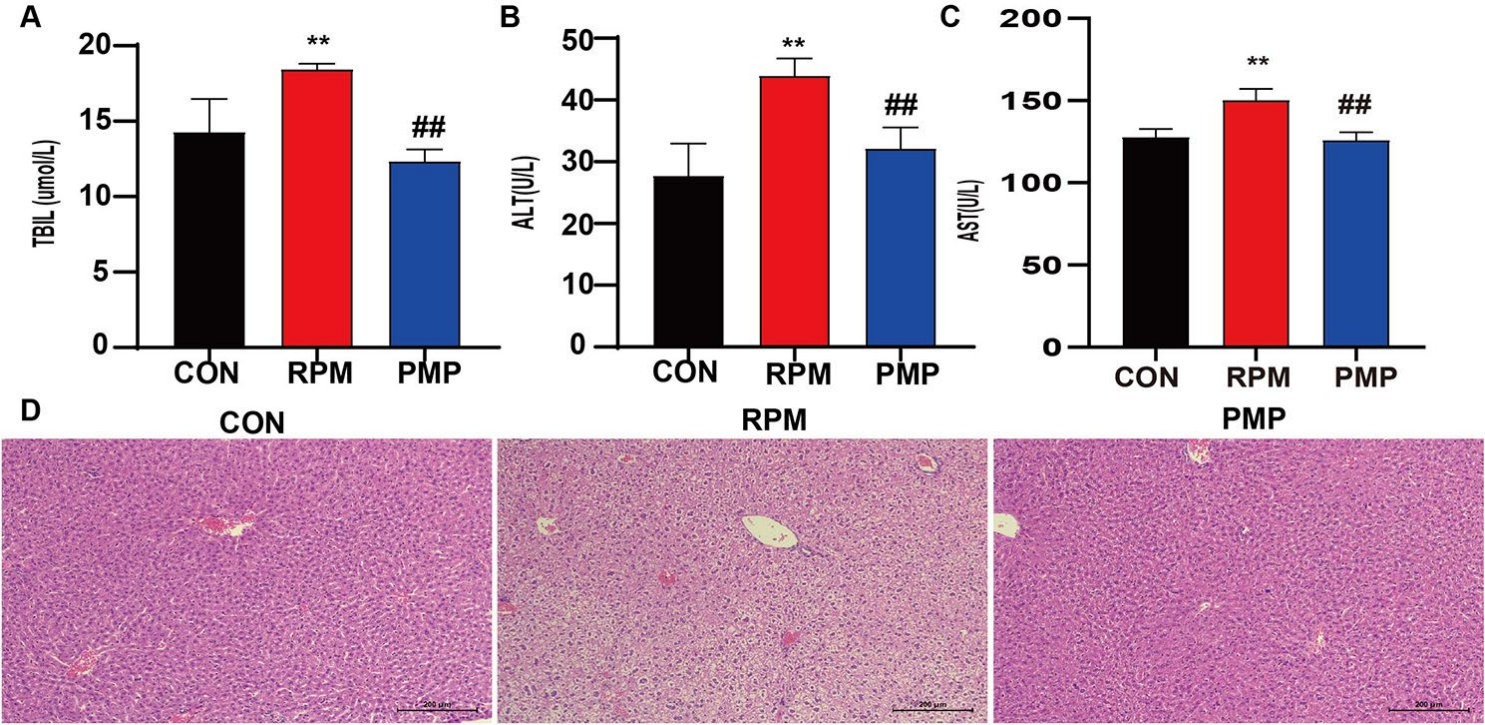
Increased liver toxicity was observed in the RPM group compared to that of the PMP group. **A-C**: Serum TBIL, ALT and AST level (*n* = 6). ^**^P<0.01 vs. the CON group; ^##^*P*<0.01, vs. the RPM group. D: HE staining was used to evaluate the morphological alternation of mice treated with RMP or PMP (*n* = 6)

To evaluate the differential adverse effects of RPM and PMP exerted on the liver, C57BL/6 mice were continuously gavaged with 9 g/kg RPM or PMP for 30 days, and biochemistry analysis along with morphological change was performed. Compared to those of the control group, the levels of AST, ALT and TBIL in the serum of the RPM group increased significantly, while the above indicators did not change significantly in the PMP group (Fig. 1A-C). Additionally, punctate necrosis, inflammatory cell infiltration and structural destruction can be observed in the RPM group. These morphological changes can be significantly attenuated after processing (Fig. 1D). These results suggested that the hepatotoxicity induced by RPM was significantly higher than that induced by PMP.

### Aggravated damage to L02 cells was observed in the RPM group compared to that in the PMP group

**Fig. 2:**
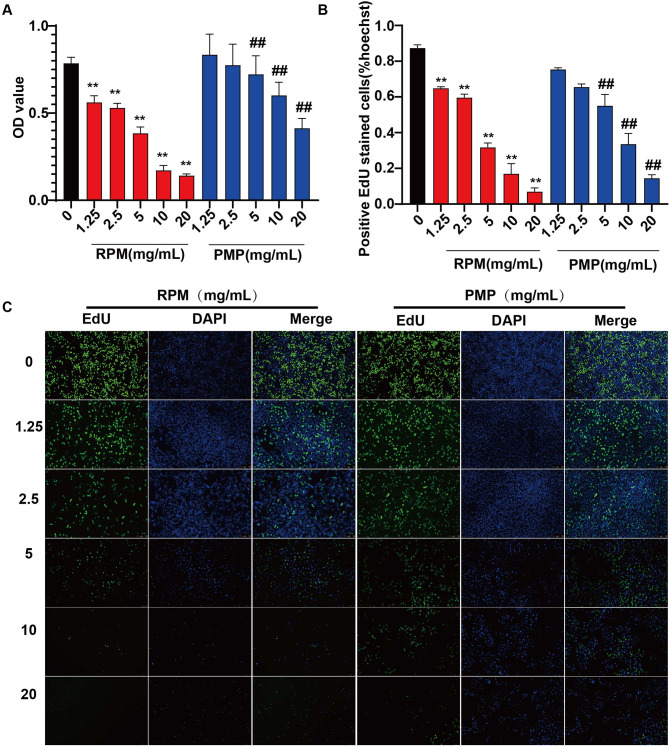
Aggravated damage to L02 cells was observed in the RPM group compared to that of the PMP group. **A**: Cell viability was measured by the CCK8 assay. ^**^P<0.01, vs. the CON group; ^##^*P*<0.01, vs. the RPM group. **B-C**: The EdU assay was utilized to evaluate the proliferation capacity of L02 cells treated with different concentrations of RPM or PMP for 24 h. ^**^P<0.01, vs. the CON group; ^##^*P*<0.01, vs. the RPM group

To evaluate the underlying mechanism of the differential hepatotoxicity exerted by RPM and PMP, we first treated L02 cells with different concentrations of RPM or PMP. The cell viability and proliferation capacity were then evaluated by CCK8 and EdU assays. Figure 2A-C uncovered that both RPM and PMP could reduce the hepatocyte viability in a dose-dependent manner, but it was much more obvious in the RPM group compared to that of the PMP group, suggesting that PM underwent processing could reduce the hepatocyte toxicity.

### Differential transcriptional expression profile of L02 cells treated with RPM and PMP

**Fig. 3:**
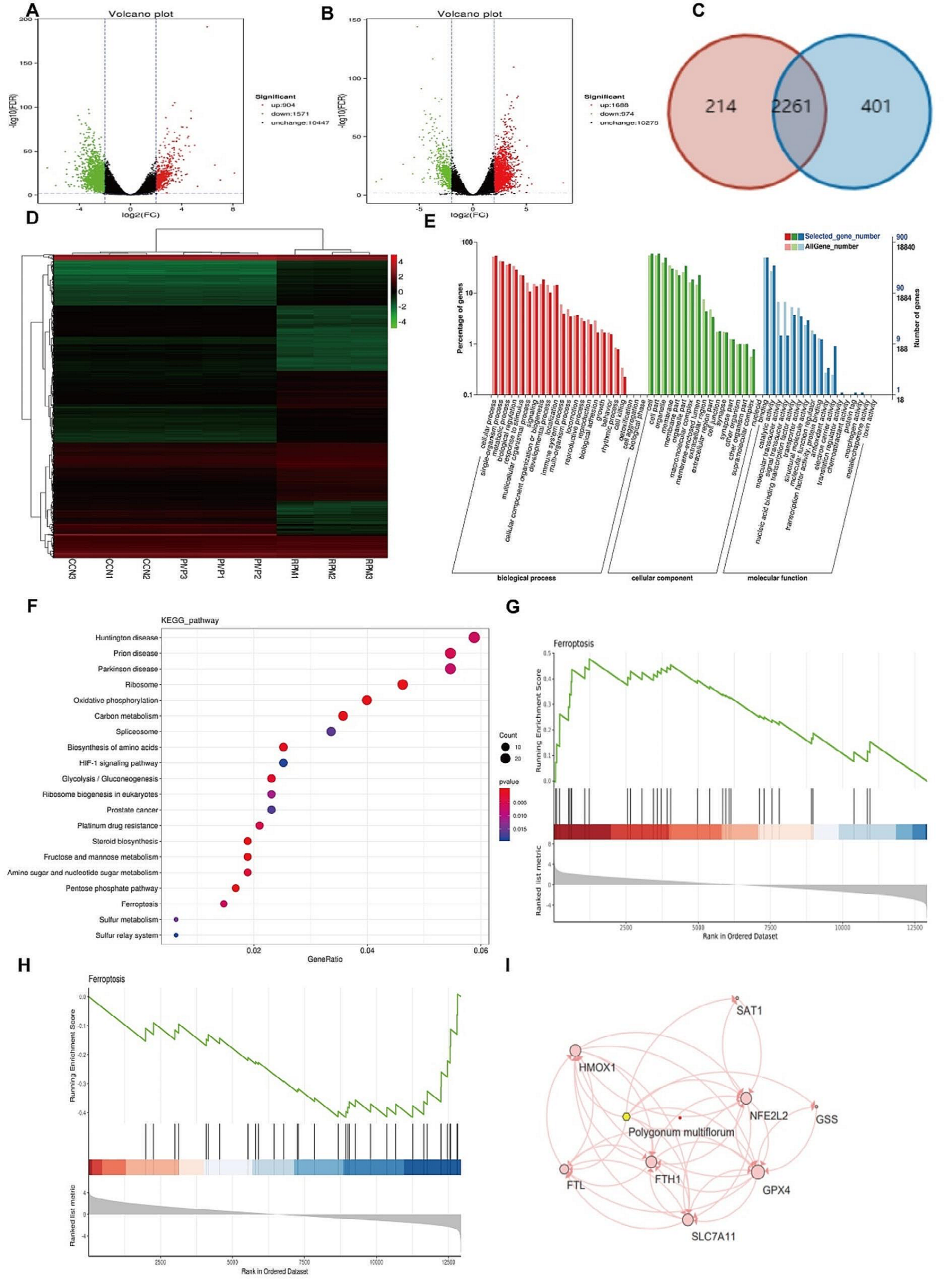
Differential transcriptional expression profile of L02 cells treated with RPM and PMP (*n* = 3). **A**: Volcano plot of the expression profile in the RPM group compared to the CON group; **B**: Volcano plot of the expression profile in the PMP group compared to the RPM group. **C**: Overlaps of the DEGs between the RPM and PMP groups. **D**: Systematic cluster analysis of intersected genes with a relative expression greater than 1. **E**: GO enrichment analysis; **F**: KEGG enrichment pathway analysis. **G**: The alternation trend of ferroptosis-related genes in the RPM group compared to the CON group; **H**: The alternation trend of ferroptosis-related genes in the PMP group compared to the RPM group; **I**: The network diagram of the overlapped genes

Next, we wanted to deeply explore the potential mechanism of differential toxicity exerted by RPM and PMP on the liver, we treated L02 hepatocytes with RPM or PMP and extracted total RNA for sequencing. Our data identified 2475 differential expression genes (DEGs), including 1571 down-regulating genes (green dots) and 904 up-regulating genes (red dots) in the RPM group compared to the CON group (Fig. 3A). Furthermore, 2662 DEGs, including 1688 up-regulating genes (green dots) and 974 down-regulating genes (red dots), were acquired compared to the RPM group (Fig. 3B). However, no significant DEGs were observed between the CON and PMP groups. Additionally, our further analysis identified 2261 overlapped genes in the identified differential DEGs between RPM and PMP group (Fig. 3C). Those intersected genes with a relative expression greater than 1 were selected for further systematic cluster analysis. A total of 962 genes with notable alternation were identified, the majority of which can be recovered after processing (Fig. 3D). Subsequently, these genes were further selected for GO and KEGG analysis. GO enrichment analysis unveiled the adverse effects of PM exerted on the liver through various biological processes, such as cell death, cell aggregation, metabolism and growth (Fig. 3E). KEGG enrichment pathway analysis showed they were primarily involved in the following signaling pathways: ferroptosis, steroid biosynthesis, and oxidative phosphorylation (Fig. 3F). In order to explore the action of PM exerted on ferroptosis, we further analyze the alternation of ferroptosis-related genes by GSEA. Our results revealed that most of the differential genes identified in the RPM group were reversed after processing (Fig. 3G-H). We next constructed a network diagram of those overlapped genes closely associated with ferroptosis, our data implicated GPX4, HO-1 and FTL were the crucial genes regulated by RPM (Fig. 3I).

### The different effects of RPM and PMP exerted on ferroptosis

**Fig. 4:**
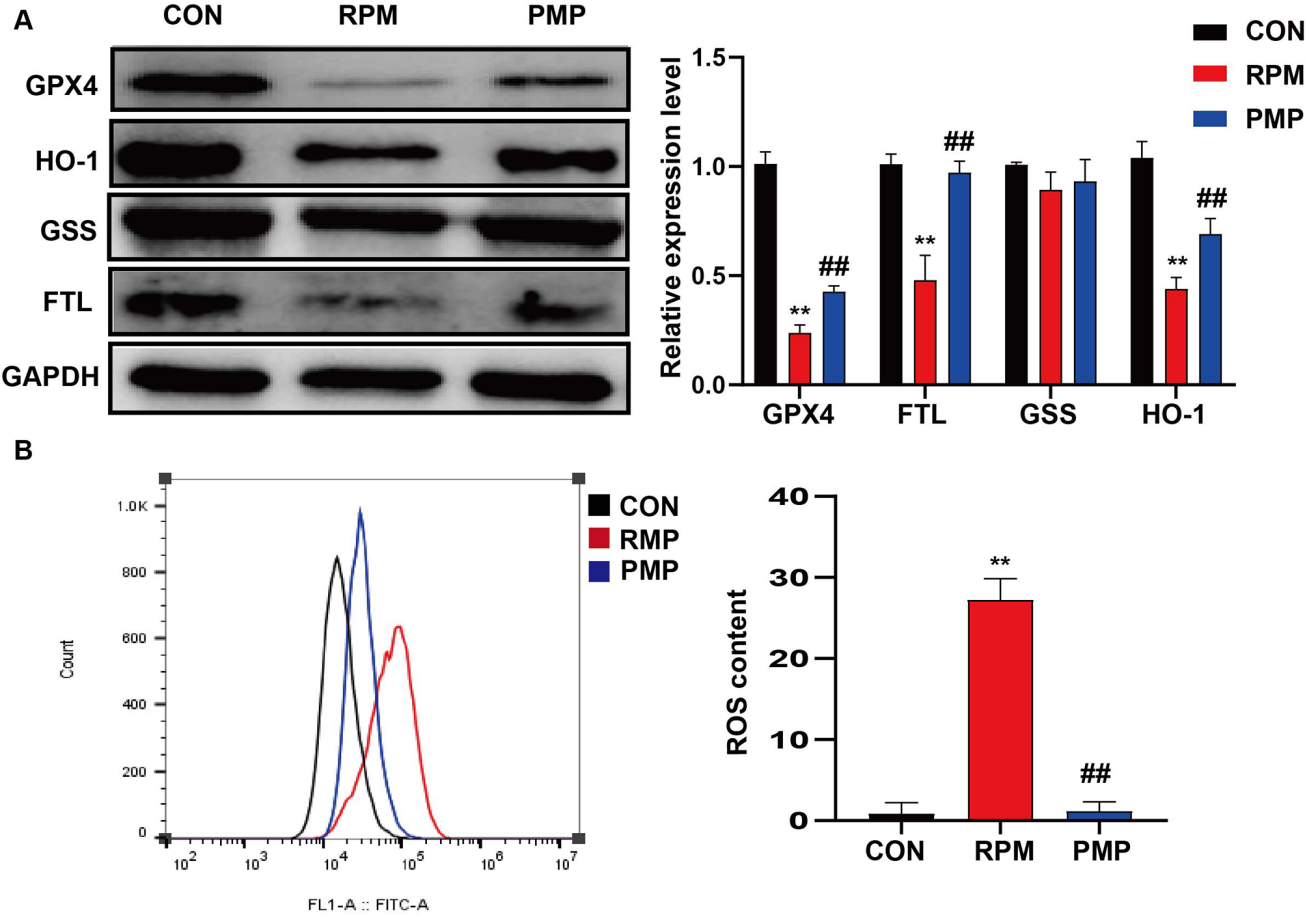
The different effects of RPM and PMP exerted on ferroptosis. **A**: GPX4, HO-1, FTL, and GSS protein levels in L02 cells treated with RPM or PMP (*n* = 3); **B**: ROS accumulation in L02 cells treated with RMP or PMP (*n* = 3). ^**^P<0.01, vs. CON group; ^##^*P* < 0.01, vs. the RPM group

Firstly, L02 cells were treated with 5 mg/mL RPM or PMP for 24 h. In order to explore the effects ferroptosis played on PM-induced hepatotoxicity, we next detected the protein levels of GPX4, HO-1, FTL and GSS by western blotting. As shown in Fig. 4, GPX4, HO-1 and FTL levels were sharply decreased, while ROS accumulation was dramatically increased in the RPM group, which can be partially reversed by processing (Fig. 4A-B). However, no significant differences were observed in the PMP group compared to those of the CON group. These results indicated that GPX4, HO-1 and FTL were the potential targets PM acted to exert adverse effects.

### Ferroptosis inhibitor reduces the hepatocyte toxicity induced by RPM

**Fig. 5:**
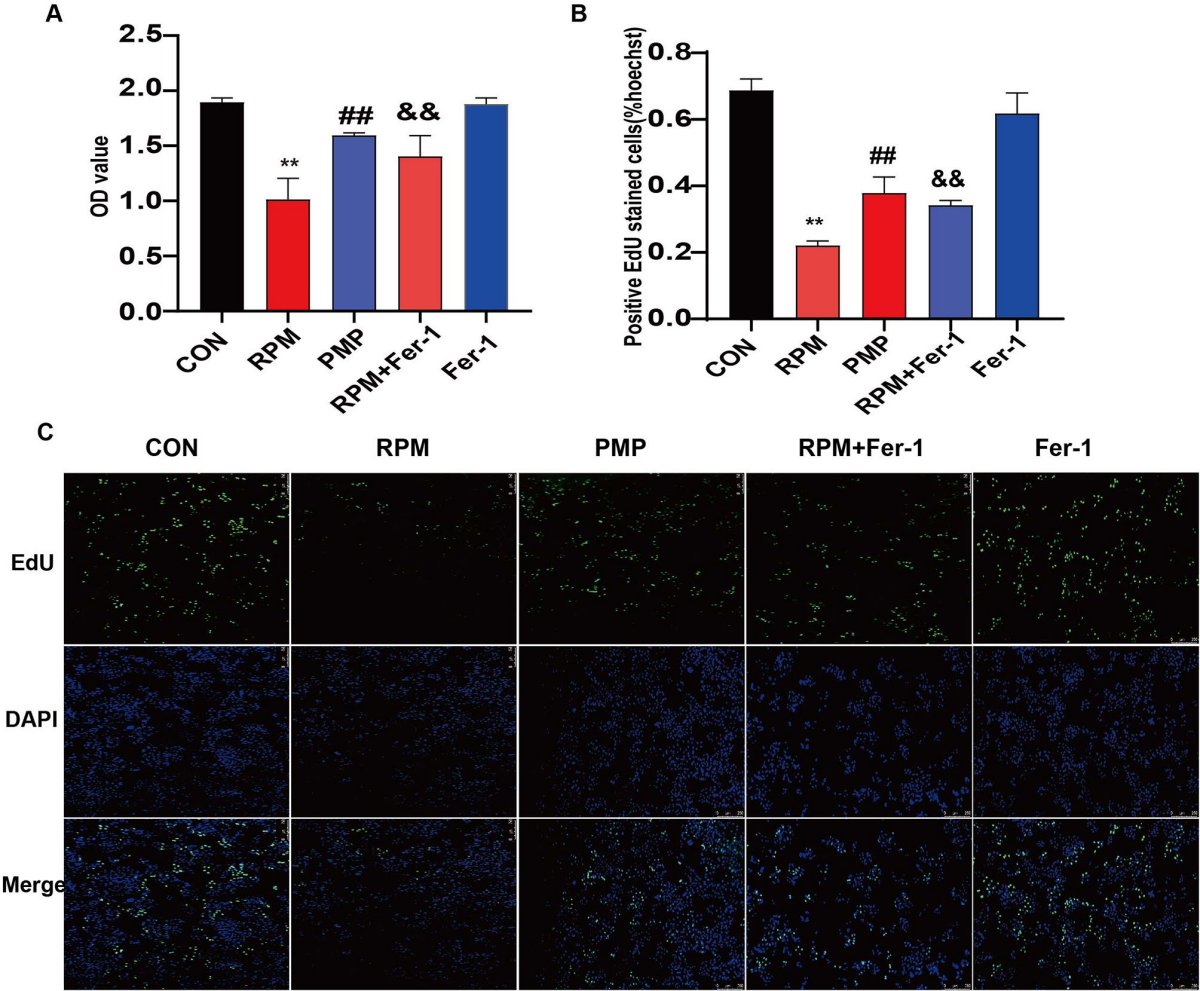
Ferroptosis inhibitor reduces the hepatotoxicity induced by RPM. **A**: CCK8 was applied to evaluate the viability of L02 cells treated with RPM plus Fer-1 (*n* = 6). ^**^P<0.01, vs. the CON group; ^##^*P* < 0.01,vs. the RPM group; ^&&^*P*<0.01, vs. the RPM group. **B-C**: EdU assay was applied to evaluate the proliferation capacity of L02 cells treated with RPM plus Fer-1 (*n* = 3). ^**^P<0.01, vs. the CON group; ^##^*P* < 0.01, vs. the RPM group;^&&^*P*<0.01, vs. the RPM group

To further confirm the effect of RMP exerted on ferroptosis, Ferrostatin-1 (Fer-1), a specific ferroptosis inhibitor, was used in our next study. Firstly, L02 cells were pre-treated with 40µM Fer-1 for 6 h, followed by the treatment with 5 mg/mL RPM or PMP for 24 h. Then, CCK8 and EdU assays were performed to examine the viability and proliferation capacity. As shown in Fig. 5A, the viability was sharply decreased in RPM-treated L02 cells, which can be significantly reversed by Fer-1. In addition, the treatment with Fer-1 (Fer-1 group) did not influence the cell viability in normal L02 cells. EdU assay analysis were highly similar to those obtained by CCK8 (Fig. 5B-C). These data suggested that RPM acted to promote liver toxicity through the regulation of ferroptosis.

### Fer-1 reduces RPM-induced hepatotoxicity by regulating GPX4 expression and ROS accumulation

**Fig. 6:**
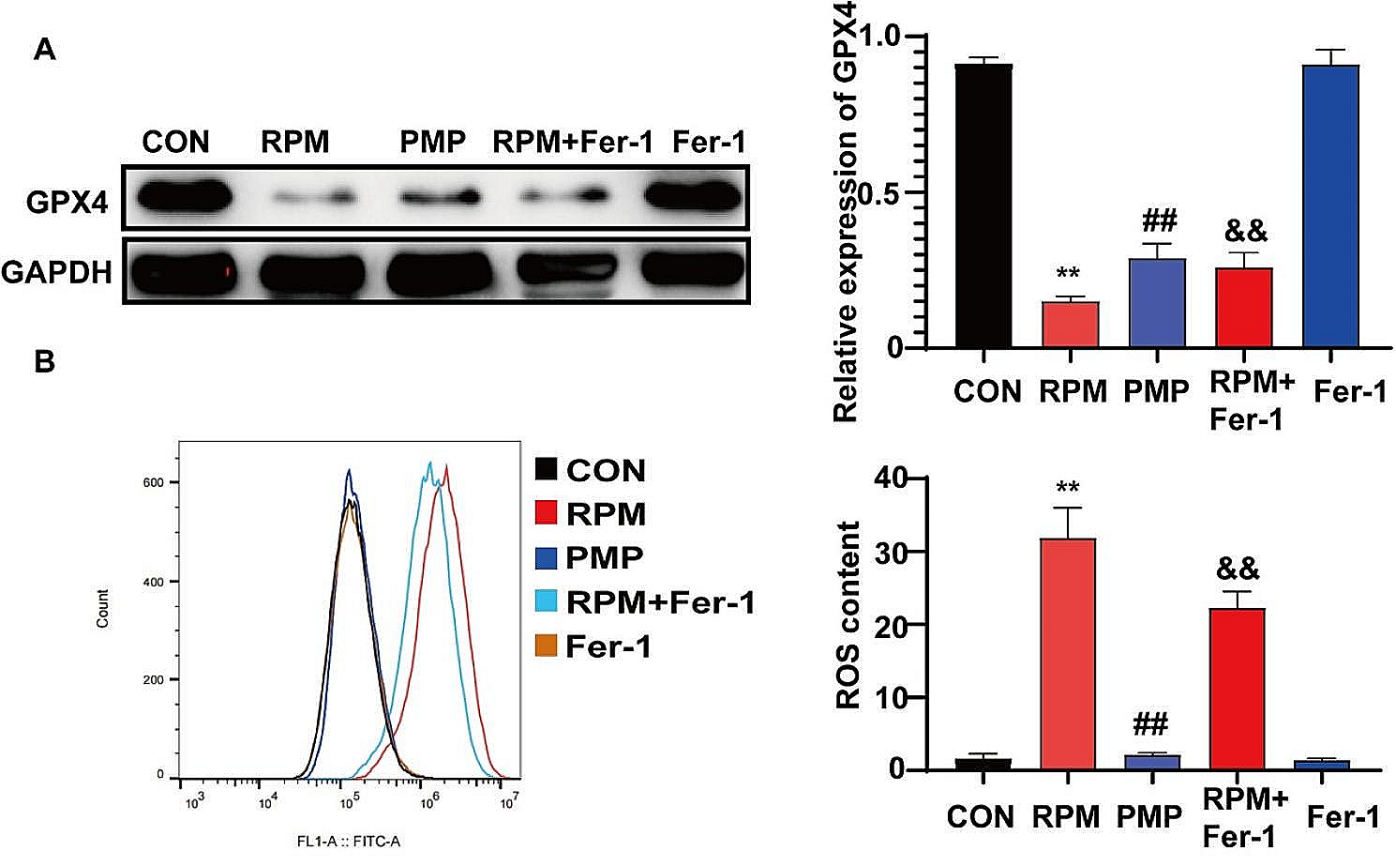
Fer-1 reduces RPM-induced hepatotoxicity by regulating GPX4 expression and ROS accumulation. **A**: GPX4 protein level in L02 cells treated with Fer-1 and RPM (*n* = 3). ^**^P<0.01, vs. the CON group; ^##^*P* < 0.01, vs. the RPM group; ^&&^*P*<0.01, vs. the RPM group. **B**: ROS accumulation in L02 cells treated with RPM and Fer-1 (*n* = 3). ^**^P<0.01, vs. the CON group; ^##^*P* < 0.01, vs. the RPM group; ^&&^*P*<0.01, vs. the RPM group

Our previous data has validated that Fer-1 could reduce RPM-induced hepatotoxicity. We next wanted to deeply explore its underlying mechanism. As GPX4 is a crucial target for Fer-1, firstly, we pre-treated L02 with Fer-1 for 6 h, followed by the treatment with 5 mg/mL RPM or PMP for 24 h. Subsequently, GPX4 protein level was detected by western blotting and ROS accumulation was investigated by flow cytometry. As shown in Fig. 6A, the level of GPX4 was dramatically down-regulated in RPM-treated L02 cells, which can be significantly reversed by Fer-1. In addition, treatment with Fer-1 (Fer-1 group) did not influence the expression of GPX4 in normal L02 cells. Furthermore, flow cytometry result revealed ROS content was significantly elevated when treated with RPM, which can be restored by Fer-1 (Fig. 6B). Together, these data suggested that Fer-1 reduces RPM-induced hepatotoxicity by regulating GPX4 expression and ROS accumulation.

**Fig. 7:**
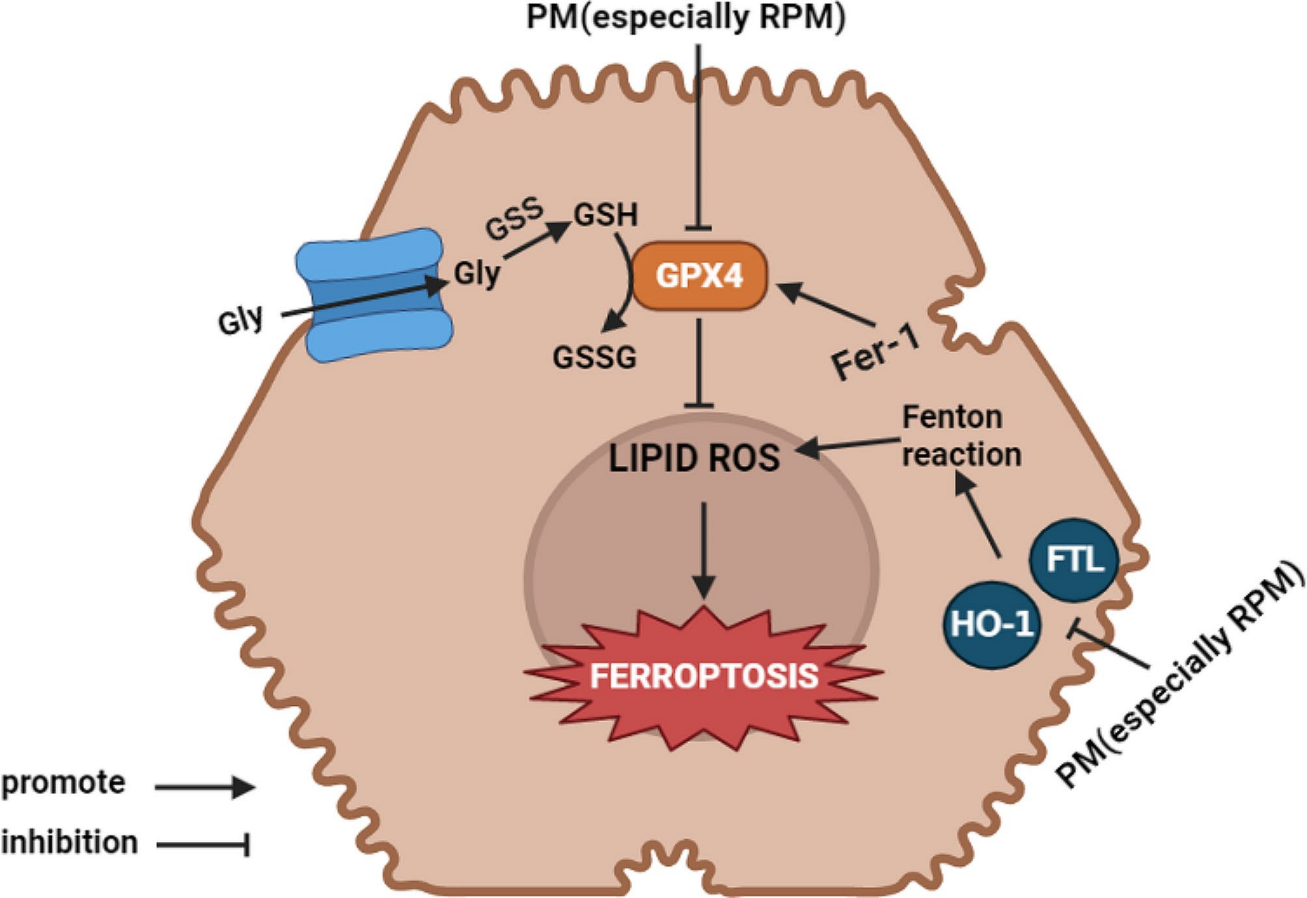
The potential mechanism of differential hepatotoxicity RPM and PMP exerted on liver damage. PM, especially RPM, could suppress the expression of GPX4, HO-1 and FTL, resulting in ROS accumulation and leading to hepatocyte ferroptosis

## Discussion

In recent years, the problem of the medicinal safety of PM has greatly limited its clinical use, and it has become the priority of current research to reduce or avoid the toxic side effects of PM while utilizing its medicinal effects [20–22]. In the present work, a significant elevation in serum ALT, AST and TBIL levels and a much more severe liver injury were investigated in mice treated with PM. These data implicated that PM could induce liver injury, which was consistent with the previous study [2].

The mechanism of liver injury caused by PM is not yet clear. In the last decade, numerous studies have indicated that PM could cause liver damage through various mechanisms. Jaeschke H et al. demonstrated that inflammation cell activation, cytokines expression and TLR-NF-kB pathway stimulation were involved in the pathogenesis of PM-induced liver injury [23]. Lin et al. revealed that RPM-induced liver injury is closely correlated with abnormal oxidative phosphatization of mitochondrial function and impaired TCA cycle signaling pathway, resulting in hepatocyte apoptosis and bilirubin metabolic disturbance [24]. Although numerous efforts have been made to elucidate the differential toxicity of liver injury by which RPM and PMP induced, the mechanism is still completely unclear. In this article, for the first time, we demonstrate the involvement of ferroptosis in PM-induced liver injury. As intrinsic hepatotoxicity usually leads to hepatic cell death, in our study, L02 human liver cell line was selected for further study. Our results revealed the viability and proliferation capacity was significantly decreased when treated with RPM. RNA sequencing data unveiled ferroptosis is deeply involved in PM-induced liver injury.

Ferroptosis is an iron-dependent form of regulated cell death characterized by ROS accumulation [25, 26]. It has been reported to be involved in the pathological process of liver damage, such as liver fibrosis and hepatocellular carcinoma [27–30]. However, the effects ferroptosis exerted on PM-induced liver toxicity is still a virgin field. In our work, the involvement of ferroptosis in RPM- or PMP-induced hepatotoxicity was investigated by RNA sequencing and verified by in vitro study. Our results revealed that RPM exposure could significantly decrease the viability and proliferation capacity of L02 cells and increase ROS accumulation. All of these side effects could be reversed by processing or Fer-1. Taken together, these in vitro data validated the higher toxicity of RPM and provided convincing evidence that hepatocyte ferroptosis was involved in RPM-induced liver damage.

Lipid hydroperoxides are regarded as crucial intermediates in the lipid peroxidation process [31]. GPX4 acted to convert lipid hydroperoxides to lipid alcohols, thus preventing Fe^2+^-dependent formation of ROS [31–33]. Suppression of GPX4 function contributed to lipid peroxidation and induced ferroptosis [13, 34, 35]. Our results uncovered the GPX4 level was decreased while ROS accumulation was increased in L02 cells with RPM exposure, which can be restored by Fer-1 or processing. These data suggested that GPX4-mediated ferroptosis is one mechanism of differential liver damage induced by RPM.

HO-1 and FTL is another important regulator of ferroptosis [36, 37]. HO-1 is a cellular inducible oxidative stress regulator acts to oxidize heme to carbon monoxide and free iron [38]. Its effects on ferroptosis have been reported to be controversial. Several researches revealed HO-1 elevation protected cells from ferroptosis by inhibiting oxidative stress [11, 39–41]. Ferritins were the most important proteins contributing to store large quantities of iron, thus exerting a central role in modulating cellular iron metabolism [26, 42, 43]. FTL, the light-chain of ferritin, is responsible for intracellular iron storage. It is also reported to be involved in ferroptosis [44, 45]. In our study, HO-1 and FTL expressions were significantly down-regulated in L02 cells treated with RPM, which can be partially reversed by processing, suggesting HO-1 and FTL were another mechanisms of the differential toxicity induced by RPM and PMP.

Emerging evidence has shown that PM-induced liver injury is the result of direct toxicity from the ingredients or reactive metabolites of PM. Anthraquinones, stilbenes and catechins or their derivatives in PM are the main controversial potentially toxic ingredients [21]. 2,3,5,4’-Tetrahydroxystilbene-2-O-β-glucoside (TSG) and combined anthraquinones such as emodin-8-O-β-D-glucoside and physcion-8-O-β-D- glucoside are dominant in RPM, while TSG, emodin and physcion are the main components of processed products [46, 47]. It has been reported that the content of TSG is decreased by ∼60%, whereas the content of emodin is increased by > 30% [2]. Li et al revealed that 3,4,5-trihydroxybenzoic acid, emodin, and 1,8-dihydroxy-3-methoxy-6-methylanthraquinone emodin 3-methyl ether in processed polygonum multiflorum were increased with the increase of processing time, but the content of TSG decreased with the increase of processing time [48]. In addition, a comparative analysis between RPM and PMP suggested 16 components may have different degrees of hepatotoxicity, including dianthrones, anthraquinone glycosides, stilbene glycosides and flavanol [49]. Therefore, we speculate that the differential hepatotoxicity between RPM and PMP may be associated with the difference in content in anthraquinone glycosides and stilbene glycosides.

In summary, in our study, we explore the potential mechanism of differential hepatotoxicity between RPM and PMP. Our results validated RPM presented with a higher toxicity than PMP. In our further study, we investigated that ferroptosis is a central regulator of PM-induced liver injury, since its inhibitor functioned to restore the viability and proliferation capacity of L02 cells. We also found that RPM-induced ferroptosis through various pathways (Fig. 7). Due to the different components in RPM and PMP, we speculated that the differential hepatotoxicity between RPM and PMP attributed to the content difference in anthraquinone glycosides and stilbene glycosides. We believe that transcriptomic studies on animal liver tissues are more convincing than studies in cell lines. The present study was conducted on the basis of previous experiments, which verified the hepatotoxicity of polygonum multiflorum only at the animal level, and due to insufficient samples of experimental animals, transcriptomic studies and further studies on ferroptosis induced liver injury were conducted only at the cellular level, and no in-depth studies on ferroptosis induced liver injury were carried out at the animal level. We believe that detect iron ion content in liver tissue homogenate, electron microscopy performed to observe the morphological structure of mitochondria in animal liver or hepatocytes can make the study more perfect and we will add them in further study. Actually, this is one of the limitations of the present study.

### Electronic supplementary material

Below is the link to the electronic supplementary material.

Supplementary Material 1

## Data Availability

All sequencing data are available through the NCBI sequence read archive under the accession number PRJNA956832(PRJNA956832 - SRA - NCBI (nih.gov)). Other datasets used and/or analyzed during the current study are available from the corresponding author on reasonable request.

## Abbreviations

CCK8: Cell-Counting-Kit-8
ROS: reactive oxygen species
ALT: aspartate aminotransferase
AST: alanine transaminase
TBIL: total bilirubin
GPX4: glutathione peroxidase 4
HE: Hematoxylin-eosin
DEGs: differential expression genes
