# Supplementary material for: Study on the differential hepatotoxicity of raw polygonum multiflorum and polygonum multiflorum praeparata and its mechanism
Source: BMC Complement Med Ther. 2024 Apr 17;24:161. doi: 10.1186/s12906-024-04463-9 (PMC11022370; doi:10.1186/s12906-024-04463-9)
Supplement: Supplementary file 1 — Supplementary Material 1 [file 12906_2024_4463_MOESM1_ESM.docx]

Original Figure 4A

1. GPX4


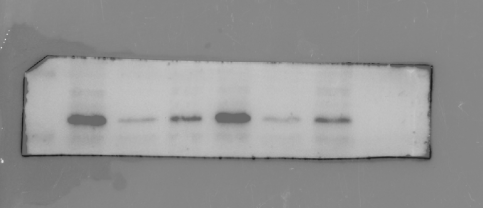


CON

RPM

PMP

PMP

RPM

CON


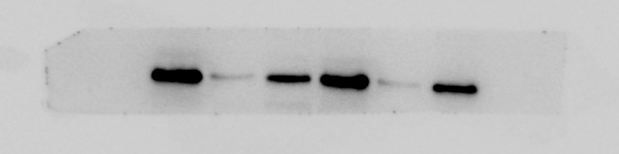


CON

RPM

RPM

CON

PMP

PMP


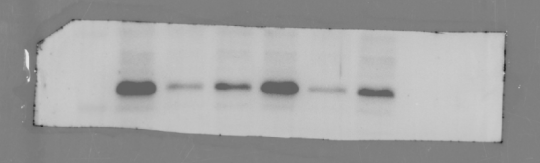


PMP

RPM

RPM

CON

CON

PMP

2. HO-1


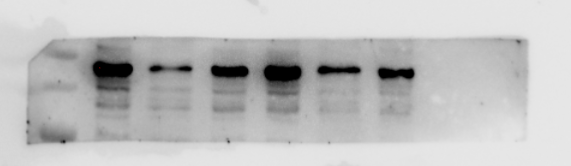


RPM

CON

CON

RPM

PMP

PMP


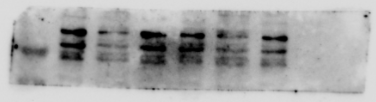


RPM

RPM

CON

CON

PMP

PMP

3. GSS


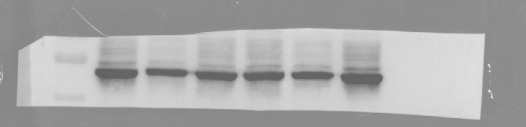


CON

RPM

RPM

CON

PMP

PMP

4. FTL


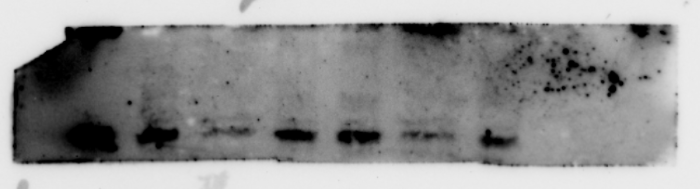


CON

CON

RPM

RPM

PMP

PMP


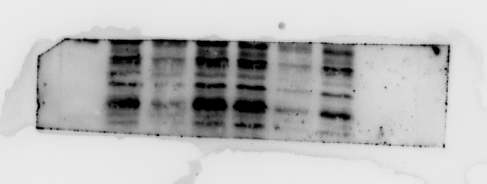


CON

CON

RPM

RPM

PMP

PMP


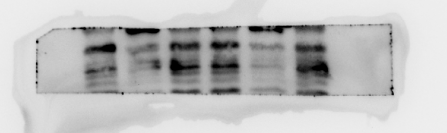


CON

CON

RPM

RPM

PMP

PMP

5. GAPDH


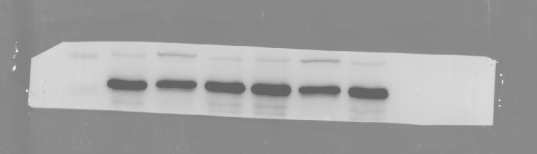


PMP

RPM

CON

CON

RPM

PMP

Original Figure 6A

1. GPX4


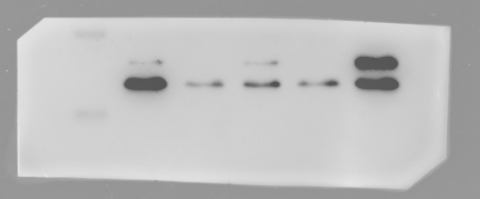


FER-1

RPM+FER-1

CON

RPM

PMP


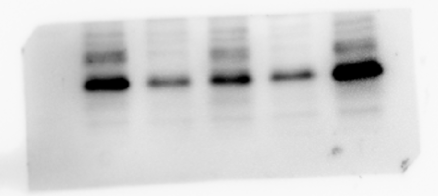


CON

FER-1

RPM+FER-1

PMP

RPM


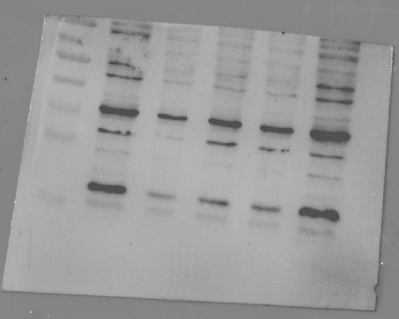


FER-1

RPM+FER-1

PMP

RPM

CON

2. GAPDH


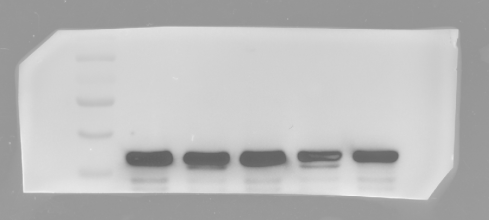


RPM

FER-1

RPM+FER-1

CON

PMP
